# Bacteriophages: Protagonists of a Post-Antibiotic Era

**DOI:** 10.3390/antibiotics7030066

**Published:** 2018-07-27

**Authors:** Pilar Domingo-Calap, Jennifer Delgado-Martínez

**Affiliations:** 1 Department of Genetics, Universitat de València, 46100 Burjassot, Valencia, Spain; jendel@alumni.uv.es; 2 Institute for Integrative Systems Biology (I2SysBio), Universitat de València-CSIC, 46980 Paterna, Valencia, Spain

**Keywords:** bacteriophages, phage therapy, antibiotic resistance, phage display, enzybiotics

## Abstract

Despite their long success for more than half a century, antibiotics are currently under the spotlight due to the emergence of multidrug-resistant bacteria. The development of new alternative treatments is of particular interest in the fight against bacterial resistance. Bacteriophages (phages) are natural killers of bacteria and are an excellent tool due to their specificity and ecological safety. Here, we highlight some of their advantages and drawbacks as potential therapeutic agents. Interestingly, phages are not only attractive from a clinical point of view, but other areas, such as agriculture, food control, or industry, are also areas for their potential application. Therefore, we propose phages as a real alternative to current antibiotics.

## 1. Introduction

Viruses can infect all types of cells, including bacteria and archaea [1]. Specifically, bacteriophages (phages) are natural killers of bacteria and they were discovered a century ago by Frederick Twort and Félix d’Hérelle, independently [2,3]. In 1915, Twort thought that pathogenic bacteria required an essential substance to grow [4]. By analyzing in detail cultures of *Staphylococcus* sp. from vaccinia virus vaccines, he observed bacteria-free regions in the culture. Although he was unaware of what kind of substance produced those halos, after observing it under the lens, he confirmed that it was bacterial debris and defined it as a bacteriolytic agent [5]. In 1917, Félix d’Hérelle designated as “bacteriophages” some entities that were able to lyse bacterial cells after examining the effect of phages against *Salmonella gallinarum* in the feces of chickens [6,7,8]. In addition, d’Hérelle was the first to apply phages as a therapy to successfully treat children with severe dysentery.

In 1923, d’Hérelle and his assistant George Eliava established the George Eliava Institute of Bacteriophages, Microbiology, and Virology (Eliava Institute) in Tbilisi, present-day Georgia. During the Second World War, the Eliava Institute’s experts provided combinations (cocktails) of different phages to soldiers, especially to treat wounds, gangrene, and diseases, such as cholera. Nowadays, the Eliava Institute carries out clinical trials with patients from all over the world, which often result in high success rates after treatment. Different phage applications in fields, such as human therapy, illness prevention, veterinary, environmental control, and food safety are investigated there [9]. This institute is responsible for the identification of more than 4000 phages, and a large number of phage-related studies have been done there. Because of this, the Eliava Institute is nowadays an important reference center for phages. Due to the discovery of antibiotics and the widespread use of penicillin in the 1940s, the use of phages fell out of favor, and, as a consequence of the Second World War, they were quickly reduced to only being used in Eastern countries, which had no access to antibiotics. Therefore, only Eastern countries (in particular, those of the former USSR), were (and are) using phages to treat bacterial infections, such as *Salmonella* and *Shigella* diseases, among others [10].

In spite of the rapid success of antibiotics, the emergence of multiresistant bacteria is a general concern. Nowadays, some bacterial strains are resistant to almost all available antibiotics. Routinely, surgical interventions can lead to serious complications due to the emergence of resistant bacterial strains that cannot be treated with conventional antibiotics [11]. Regarding the origin of this resistance, horizontal genetic transfer has been thought of as a key factor in the acquisition of antibiotic-resistant genes [7]. Moreover, spontaneous mutations can also occur in some genes under the action of antibiotics, and therefore contribute to its emergence [12]. High mutation and gene flow rates allow for bacteria to evolve quickly under the strong selection pressures that are exerted by antibiotics. In addition, the use of broad-spectrum antibiotics and their misuse promote this problem [13]. As a consequence, bacteria have developed several mechanisms to prevent antibiotic function, such as changes in receptors through enzymes or mutation, removal of the antibiotic by membrane pumps, or antibiotic modification to escape its effect [12,13,14]. For these reasons, it is important to propose alternative methods to fight against bacterial resistance. Here, we demonstrate how bacteriophages can be useful in this battle, and a wide variety of interesting phage applications are also reviewed. 

**Figure 1:**
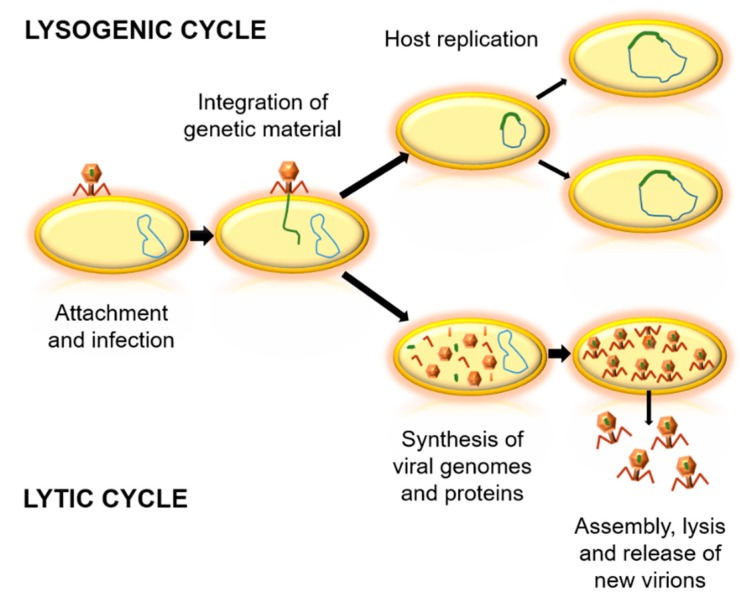
Biological cycles of phages. Firstly, the virus binds to the bacterial cell and injects its genetic material. In the lysogenic cycle, the integration of viral genetic material into the genome of the host occurs, and the bacterial cell replicates without producing virions. In the lytic cycle, viral genetic material is replicated and viral proteins are synthesized. Then, an assembly of virions is achieved, followed by the lysis of the bacteria and the release of new virions.

Bacteriophages can be classified according to their genome, morphology, biological cycle, or the environment where they live [15,16,17]. Concerning the biological cycles of phages, there are two main types—lytic and lysogenic cycles (Figure 1). The lytic cycle implies bacterial death, which generates virion output. For this, they take advantage of the replication system of the host cell, and when their proteins and viral components are formed, they induce cell lysis [18,19]. In contrast, lysogenic cycles are based in the integration of the genetic material of the phage into the genome of the host cell. At the end of the replication cycle, no new virions are obtained, but bacterial cells with phage genetic material are created, as temperate phages [18]. Due to the variability between phages, it is important to determine which are the most appropriate bacteriophages for each potential application [20].

## 2. Phages in the Biosphere

Viruses are ubiquitous in the biosphere and can be found in all environments, being the most abundant biological entity [21]. It is believed that there are 10^31^–10^32^ virions in the biosphere, approximately distributed as 2.6 × 10^30^ virions in soils, 1.2 × 10^30^ virions in the ocean, 3.5 × 10^30^ virions in the oceanic subsurface, and 0.25–2.5 × 10^31^ virions in the terrestrial subsurface [22]. In addition, significant viral quantities have been found in extreme environments, with between nearly 9.0 × 10^6^ and 1.3 × 10^8^ virions mL^−1^ in sea ice and approximately 5.6–8.7 × 10^10^ virions cm^−3^ in algal flocks [16]. Other extreme conditions in which viruses have been found include high-temperature environments (thermal waters, geothermal springs, volcanoes, hydrothermal vents, etc.), cold environments (lakes of polar areas, sea ice, etc.), and hypersaline zones [22]. Although few studies have focused on their presence in soils and the rhizosphere, around 1.5 × 10^8^ virions g^−1^ are estimated to be present there [16]. Bacteria can be found in almost any environment, such as seawater, fresh water, and soils [23], so phages are expected to be found in any place where a host is located (Table 1). In addition, some of them may be specifically localized, whilst others can be widely distributed throughout the biosphere [24]. Indeed, only around 6000 different bacteriophage species are known, hiding a great diversity that is still unknown [25]. It is theorized that, in the ocean, there are at least 10^7^ phages mL^−1^ [17], and the number of soil phages could be as great as 10^8^ virions g^−1^ [26], representing a large proportion of the total amount of viruses in the biosphere.

Phages and bacterial cells have been coevolving for a long time, showing dynamic interactions between them [27]. Experimentally, it has been shown that coevolution between phages and bacteria can increase the rate of molecular evolution. This has been studied in *Pseudomonas fluorescens* SBW25 infected with the phage Φ2, and it has been shown that not all genes evolve equally in the phage. Interestingly, those that evolve quickly are related to the infection of the bacterium, coding for proteins that are related to host attachment [28]. In addition, coevolution leads to the maintenance of bacterial diversity and is responsible for changes in the physiology, abundance, abilities, and virulence of bacteria [29]. Similarly, phages are also influenced by their interaction with bacteria, especially in their defense strategies [30]. Although phages are highly specific, some of them show a wide host range. Moreover, due to phage-bacteria interactions, phages can participate in the biogeochemical cycles of biotic and abiotic environments. When bacterial lysis occurs, bacteria debris remains in the medium, being a nutritional source, and carbon, nitrogen, and phosphorus cycles are enriched or modified [31]. Therefore, phages play an important role in their environment. For example, they participate in nutrient acquisition in marine ecosystems and improve carbon transfer through phage lysis [32]. 

As with bacteria, we can find phages living in higher organisms, mostly in the digestive tract, vagina, respiratory and oral tract, skin, and mucosal epithelium, forming the so-called “phageome” [33]. Due to the great diversity and quantity of phages in the body, it is possible that they participate in human homeostasis. For example, gut phages are lytic and temperate, and both types are important for avoiding bacterial imbalance. It is suggested that the introduction of viruses in the gut occurs during the first four days of life and that they undergo changes with the development of the body [34].

Thanks to metagenomics, it is possible to determine phage variability and their abundance in each environment. Epifluorescence microscopy and transmission electron microscopy can help to identify new phages [22]. Before these techniques, counts were made in bacterial cultures and by obtaining plaque forming units (PFU), which may lead to difficulties mainly because not all bacteria are cultivable under artificial conditions, and not all phages make lysis plaques [35]. For these reasons, the real abundance and diversity of phages are higher than observed.

Phages can also be found in artificial places or infrastructures that humans inhabit, such as hospitals, showing that natural places are not the only source of phages [36]. Hospital sewage is an especially good reservoir of phages. As explained by numerous articles about multiresistant bacteria, phages that were isolated from the wastewater of medical centres are used in applications against resistant bacteria that cause diseases. Additionally, clinical materials or medical devices can be a source of phages [37,38]. 

Remarkably, wastewater treatment plants (WWTPs) are the habitat of many types of microorganisms, which makes them interesting considering that many interactions among bacteria and phages take place in them. More than 1000 different types of viruses have been found in WWTPs and a large proportion of them are bacteriophages. The water of WWTPs undergoes several debugging and cleaning processes to obtain potable water, and these physical and chemical methods manage to eliminate many bacterial cells, although they usually fail to remove phages. Therefore, this affords an opportunity to isolate bacteriophages [39]. 

Accordingly, phages are a ubiquitous ecological solution to develop new treatments against bacteria, although further studies should be done to better understand the relationship between them and bacteria before their application. 

## 3. Potential Application of Phages

Phages should be considered as great potential tools due to their multiple benefits. Since their discovery, phages have been used as models to understand fundamental genetic processes and as great tools in molecular biology. Phage products, such as ligases, polymerases, or recombinant phages, are commonly used in research laboratories. Here, we place an emphasis on the potential application of phages against pathogenic bacteria. The emergence of multidrug-resistant bacteria has led to the need for new treatments. To this end, we assess how phages can help to overcome this critical situation by coming up with potential applications of phages that may be of interest in different areas. Different approaches using phages are proposed, and some of the most relevant ones in the fight against bacterial resistance are described in detail. 

### 3.1. Phage Therapy

Phage therapy is based on the therapeutic use of phages to treat pathogenic bacterial infections [40]. Lytic phages are preferably chosen in phage therapy for two main reasons. Firstly, because lytic phages will destroy their host bacteria, whilst temperate ones will not. Secondly, because temperate phages can transfer virulence and resistance genes due to their life cycle, in which the genome of the phage is integrated into and replicates together with the bacterial genetic material [8,19]. The intrinsic characteristics of lytic phages, such as high host-receptor specificity and bacterial cell lysis to release virions, make them highly suitable for clinical applications [7,41]. Some remarkable features of the use of phages include their short replication time and their ability to obtain a large number of viral progeny only in their specific hosts, the specificity to prevent damage in nonpathogenic bacteria (they are ecologically safe and have no known side effects), and their fast and low-cost production. In addition, their short genomes allow for us to understand the molecular mechanisms implicated in controlling resistant cells [7]. Another significant feature of phages is their ability to coevolve with their host, in a hit-and-run response, to counteract possible resistant mutants, with higher mutation rates being described for viruses than for bacteria [30].

#### 3.1.1. Main Applications of Phage Therapy

As previously mentioned, phage therapy has been used since a century ago. However, their use is restricted to Eastern countries, which have different guidelines for clinical trials and research articles are mainly published in Russian or other non-English languages. Because of that, phage therapy is not currently used in European and North American countries. Researchers are now making efforts to follow clinical trials guidelines to use phages in clinics. An interesting European project under human clinical trial is called Phagoburn, which was funded in 2013 by the European Commission. This project is based in the use of phage cocktails for burn injuries infected with *Escherichia coli* or *Pseudomonas aeruginosa* [42].

The main application of phage therapy is its use as a therapeutic agent to eliminate pathogenic bacteria involved in disease or infection as well as those that form biofilm. Another interesting approach is the use of phages as a preventive disinfectant, especially in medical areas or clinical devices. Additionally, current technology or the combination of phages with other techniques can improve these clinical applications. Despite the effectiveness of a single type of bacteriophage against a bacterial strain due to its high specificity, phage cocktails are an interesting strategy to solve issues relating to resistance and a low range of action [43]. They are normally composed of different phages that attack different bacterial strains or species. In this way, phage cocktails can play a decisive role in biofilms by allowing for the phages’ effects to last longer by delaying the emergence of resistance to all the phages that are part of the cocktail. Furthermore, it has been proven that these cocktails have other benefits, such as decontaminating food by removing *E. coli*, *Salmonella enterica* or *Listeria* [44].

Outside medicine, phage therapy plays an important role in other fields, such as food production and cattle raising. Phages are useful to ensure food safety because they allow for the removal of bacterial infections in animals and thus prevent the consumption of contaminated food. Some interesting examples are the use of phages to control typical food infections that are caused by *Salmonella* (salmonellosis), *Campylobacter* (campylobacteriosis in poultry), *Listeria monocytogenes*, or *E. coli* [2].

#### 3.1.2. Benefits and Drawbacks of Phage Therapy

Bacteriophages present many benefits that make them excellent tools to treat bacterial diseases and to contribute to the fight against the emergence of bacterial resistance. One of the greatest concerns regarding antibiotics is their side effects, since they can damage the microbiome, which is related to many types of imbalances or diseases. In this regard, the specificity of phages can solve this problem, since they will only replicate inside their specific host (phages cannot infect eukaryotic cells). In contrast to antibiotics, phages can proliferate quickly inside the host (and only if they find their host), can be administered in small doses and with long intervals of time between them, and they are removed once their population is eliminated [2,23]. The action of phages inside the host is very specific, as phage replication only occurs inside bacteria. On the contrary, antibiotics are less precise and they reach more areas without the presence of bacteria in the organism [11]. Another benefit of phages is that they can be used in difficult-to-reach parts of the body, such as treating central nervous system infections, which commonly poses a serious problem [8]. A remarkable feature of phages is that they can evolve, whilst antibiotics are static substances that cannot change even if their environment changes. Another interesting feature of phages is their isolation and production costs, as previously mentioned. The cost of antibiotic production is high, both economically and because antibiotics are not natural and have to be synthesized in a laboratory [23]. 

It is worth noting that the great specificity of phages is both an advantage and a limitation of phage therapy. Phages avoid damage to the microbiome, but prior to their application, it is necessary to determine in vitro which bacteria are causing the disease. This can be a difficult process because identification must take place quickly in order to apply the treatment to the patient [45,46]. A way to solve this setback is the use of phage cocktails, as this widens the range of action [2]. However, it is possible that in vitro and in vivo phage behavior may be different, and as a result of the lack of in vivo studies, their effectiveness cannot be fully assured [47]. 

The pharmacology of phages can be very complex, for both the action of phages inside the body (pharmacodynamics) and the body’s function on the phages (pharmacokinetics) [33]. In phage therapy, the interactions between phage and bacteria are related to pharmacodynamics. Regarding pharmacokinetics, it is believed that this is linked to the density of phages within the hosts. In the event of a small bacteria population, a large dose of phages must be used in order for them to replicate faster than bacteria. Furthermore, if the bacterial density is small, the phages might not replicate quickly enough and they will not perform the desired action [48]. This can depend on the phage dose, which in turn depends on the bacterial density, the size of phage particles, and the phage virulence, as the more virulent the phage, the better it will attack its host. The resolution of this point is based on a virulent phage with a great burst size (producers of large progenies in a short time) and that is specifically administrated at the infection site.

In addition, it is possible that phages or their products can be recognized by the immune system and induce immune responses. Nevertheless, phage lysis is usually faster than the action of neutralizing antibodies phages. However, some researchers suggest that it is possible that an immune response occurs, owing to the products and enzymes that are released from bacterial lysis. Noteworthy, recent studies showed that phage T4 is highly immunogenic and can be used as potential vaccine candidates [49]. In addition, in many cases immune responses can be avoided by modifying the mode of phage administration [33]. The immune mechanisms that detect phages and subsequently take action are not well understood. Thus, it is necessary to investigate these matters to evaluate phages’ effect on the body. On the other hand, different studies are in agreement that the application of phage therapy has no direct consequences on the patient [50]. Despite the fact that phage safety must be confirmed, phages are consumed indirectly by means of fermented foods, breathing, or every time we accidentally drink sea water. For that reason, it seems that bacteriophages do not pose a potential risk [51]. Apart from the route that phages naturally use to arrive in their bacterial host, there are new strategies to improve the lifespan of bacteriophages in an organism. Biomaterials that do not interfere in phage activity should be used, as liposomes or capsules around phages that are made of alginate are accompanied by different ions. One question that needs to be dealt with is the physical limitations of these structures, and the most appropriate way of encapsulating phages must be chosen according to their future function [52]. 

Above all, the most urgent point that should be solved is the scarcity of basic information related to doses, forms of administration, protocols, and the correct mode of application of this therapy in the specific case of each phage [2,45]. This question, together with the difficulties of patenting phages (since they are natural entities), is an impediment for pharmaceutical companies to accept this therapy [42]. Legal regulations must be established to define limitations and the safe use of phage therapy. Lastly, ethical and social acceptance of phage therapy is a great impediment, since it is difficult to believe that viruses not only are not dangerous for humans, but that they have also the potential to treat diseases.

#### 3.1.3. Emergence of Bacterial Resistance against Phages

**Figure 2:**
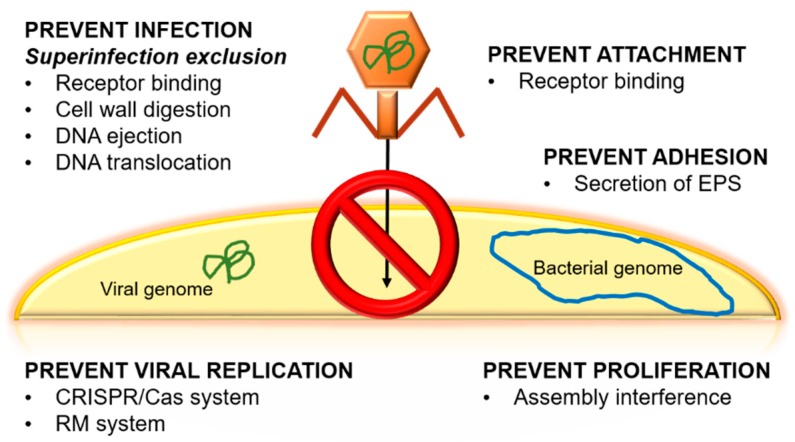
Principal natural mechanisms to prevent viral infection in bacteria. EPS: extracellular polymeric substances, CRISPR/Cas: Clustered Regularly Interspaced Short Palindromic Repeats/Caspase, RM: restriction modification.

Another controversial topic is the emergence of bacterial resistance against phages. Bacteria present natural mechanisms to prevent viral infection (Figure 2). Some of these mechanisms are associated with phages’ receptors, e.g., bacteria can hide, change, or even lose phage receptors [8]. Each of these mechanisms is activated in response to a stimulus, for example, receptor loss usually occurs when there is a change in the composition of the bacterium’s cell surface, as is seen in *Bordetella* spp. and *Shigella flexneri* [8,41]. If the loss of the receptor occurs, the phage cannot recognize the bacteria, and, subsequently, no new phages will be generated. This occurs, for example, in *E. coli* and *Staphylococcus aureus* as a consequence of membrane protein modifications. Some bacteria even have the ability to secrete extracellular polymeric substances (EPS), glycoconjugates, or alginates in order to prevent the adhesion of the phage to the bacteria. These secretions have been observed in *Pseudomonas* spp., which ejects EPS, and *Enterobacteriaceae*, which secretes glycoconjugates [41].

There are others systems to escape phages, such as by viral DNA removal by different methods, Clustered Regularly Interspaced Short Palindromic Repeats (CRISPR)-associated proteins, or the superinfection exclusion (Sie) system [9,41]. CRISPR is considered to be the immune system of bacteria to protect the genetic material of the cell against possible attacks of viruses or plasmids [53]. To counteract the CRISPR system, phages have been co-evolving with bacteria thanks to phage-anti-CRISPR [54]. On the other hand, Sie is based on membrane-associated proteins. These proteins interact with proteins that are related to DNA injection when the phage is bound to a membrane receptor. Through this process, it is possible to interrupt the injection of DNA and thus reduce phage virulence, preventing the infection from spreading [55]. Another process is based on the abortive infection (Abi) system, which is responsible for interfering with the replication, transcription, or translation of the phage or in virions formation [41]. This mechanism is activated when the phage has managed to enter the cell because it has evaded the restriction systems and the CRISPR system of the host. The objective of Abi is to destroy the infected cell so that it does not transmit the invasion to the rest of the cells [30]. An interesting way to increase phage fitness against bacteria and to reduce the emergence of resistance is experimental evolution. This method consists of preadapting a phage to its host in vitro for several generations. In addition, experimental evolution produces benefits for phage therapy, since the bacteria–phage coevolution over many generations can make the bacteria diminish their ability to adapt to the environment [56].

The combination of phages with antibiotics is another strategy to apply phages as therapeutic agents. It is possible to reduce resistance because phages can kill antibiotic-resistant variants and vice versa [57]. Owing to these combinations, the phage-antibiotic synergy (PAS) effect usually takes place, which consists of an increase in phage virulence as a result of the administration of sublethal concentrations of antibiotics and it can be convenient to remove pathogenic cells quickly [43]. In addition, additive effects of the components can occur and they can be beneficial. Through animal studies, it has been proven that these treatments have preventive action for bacteria resistance [45], for example, decreasing mutations that confer resistance to bacteria [46]. On the other hand, some of these antibiotics block the cellular cycle of bacteria, and as a result, bacterial cells undergo an increase in volume that facilitates the division of phages and their release at a faster pace [58]. Some studies have confirmed the effectiveness of this method, like the combination of phage SBW25φ2 with kanamycin antibiotic against *P. fluorescens* SBW25 [57]. The success of combinations depends on the target cell and phage and antibiotic types. Moreover, it is important that phages and antibiotics detect different regions of union of pathogenic bacteria to ensure the effectiveness of the treatment. These deductions arise from experiments with different phages and antibiotics to decrease *E. coli* in urinary tract infections. Specifically, the best results were obtained from the combination of phages and the antibiotic ciprofloxacin (at a sublethal concentration) against *E. coli* [57]. This synergy has been shown using cefotaxime combined with phage T4 against biofilm formations of *E. coli* [59]. Furthermore, because of the lack of information about the safety of phages, and sometimes the ignorance of phages’ effects, it is estimated that the combination of both provides more support than the use of individual bacteriophages only [45]. It is thought that this therapy can be better than phage cocktails because phages and antibiotics act in different ways inside bacterial cells, while all the phages in a phage cocktail may have similar modes of action, which might be a problem for the emergence of resistance [60]. Nevertheless, this association presents some defects. As a consequence of the PAS effect, and mainly due to antibiotics in sublethal concentrations, an SOS (emergency repair) response can arise, which consists of bacterial responses to stress as a consequence of damage to bacterial DNA, causing serious effects by increasing the antibiotic resistance. Other side effects can be the appearance of double resistant mutants, the potentiation of antibiotic resistant bacteria due to the phages preference to infect sensitive variants, or the interference between the action of the phage and the antibiotic resulting in an action that is less effective than the sum of both [45]. It is also important to know when to apply each treatment (phages and antibiotics). It seems that to prevent resistance, an intermediate interval of time between phage and antibiotic applications is better than the simultaneous application of both or their use after a very long period of time between them [60].

### 3.2. Phage-Derived Enzymes

An alternative to the use of phages against bacterial diseases is the use of phage-derived enzymes. Phages produce several enzyme types, each one being suitable for attacking specific bacteria [61]. Phage enzymes were discovered in 1986 when investigating an enzyme secreted by *Streptococcus* after being infected by a bacteriophage. The activity of this enzyme was the rupture of the cell wall and it was called lysin [62,63,64]. In nature, these enzymes are found inside phages and help them to penetrate into the host to assure phage replication, among other functions. In general, they degrade peptidoglycan, thereby producing bacterial lysis by osmotic imbalance. Lysins can be used as enzybiotics because, applied exogenously as recombinant proteins, they can remove bacteria. This approach has been used for more than 20 years, especially against Gram-positive bacteria. It was thought that Gram-negative bacteria could physically block the passage of lytic enzymes due to the presence of its outer membrane, but it was later discovered that some phage enzymes can cross this layer. Although it is postulated that these enzymes may cause unwanted immune responses, several studies have shown no serious side effects after their use [65].

Despite their success in killing bacteria, they present some problems of stability and lack of solubility, which may be solved by enzyme engineering. Sometimes, the use of recombinant enzymes is recommended. The great progress in enzyme engineering and synthetic biology, in addition to their low cost of production, makes this technique one of the most effective against antibiotic resistance in its application in clinics. Moreover, lytic enzymes are interesting in other areas, such as agriculture, food industry, diagnostics, environmental control, and bioluminescence [65].

As previously mentioned, one type of phage-derived enzyme is lysins. Within them, we can find endolysins, which are derived from the lytic cycle, and virion-associated lysins (VALs), which are implicated in the entrance to the host cell. Some of them can suffer changes upon the modifications of their host cell and are very specific to species or bacterial serotype [61]. VALs can be part of the phage tail or be inside the capsid. Once the phage is recognized by its receptors in the host, a conformational change in the phage allows entrance into the bacteria thanks to the degradation of peptidoglycan carried out by VALs. The mode of action of VALs against bacteria consists of allowing for the injection of the phage. This fact is possible thanks to the rupture of the peptidoglycan layer of the bacteria through the hydrolysis of chemical bonds. In its clinical application to treat bacterial diseases, this ability is used to induce the osmotic lysis of the bacteria [61]. Differently, endolysins must cross the cell membrane to reach the cell wall and cause lysis, since they are synthesized in the cytoplasm of infected cells at the end of the viral cycle. They are classified into canonical endolysins (the most interesting as enzybiotics) and exported endolysins. Canonical endolysins need other specific phage enzymes, called holins, which will form holes through which the endolysins will leave the cytoplasm [65]. Endolysins are useful as alternatives to antibiotics due to their bactericidal function. This ability has been proven by applying these purified enzymes on bacteria. Endolysins usually have two domains: one is catalytic active (N-terminal), whilst the other attaches to the cell wall (C-terminal). Additionally, some laboratories are creating chimeric endolysins in order to improve bacterial lysis. One example is the chimera Cpl-711, which combines the endolysins Cpl-1 and Cpl-7S from Cp-1 and Cp-7 pneumococcal bacteriophages against *Streptococcus pneumoniae* and their multiresistant strains [66]. An interesting feature of lysins is their high specificity, which reduces the probability of developing bacterial resistance, making resistance an extremely rare event [61].

Holins are proteins derived from phages acting at the end of the lytic process in order to trigger and control the degradation of the cell wall from the bacterial cells. These small membrane proteins control lysis time and are diverse. They are commonly small, have a positive and hydrophobic C terminal domain, and include one to three hydrophobic transmembrane domains by which they can be classified [67]. Membrane channels or pores are created when the concentration of holins exceeds a threshold, and then endolysins will perform their function. The canonical holins form large pores at one side of the host and locally expose the peptidoglycans to cytoplasmic canonical endolysins. Pinholins, another group, form small pores that depolarize the membrane, triggering signal-anchor-release (SAR) endolysin activation and inducing degradation of peptidoglycans in the whole cellular periplasmic space [68]. They can be combined with other enzymes to amplify the host range of endolysins. Since holins have a large strain spectrum, they are interesting against multiresistant strains such as *S. aureus* or *S. suis* [19].

Polysaccharide depolymerases are very useful due to their ability to attack carbohydrates of bacterial membranes [19,69]. Depolymerases present two different forms, as part of the phage particle that is attached to the base plate (in the phage membrane or capsid) or as a protein resulting from cell lysis after phage replication. They also differ in the mode to remove carbohydrate polymers on the membrane of the host bacteria. They are called hydrolases when they are able to hydrolyse the glycosyl–oxygen bond and turn it into a glyosidic bond. In contrast, lyases add a double linkage into uronic acid particles, thanks to the β-elimination system, after the disruption of the glycosidic linkage between a monosaccharide and the C4 of the acid. An example of lyases are the hyaluronan lyases [67]. Lyases would be an indirect way of dealing with bacteria because these enzymes act on bacteria that are encapsulated by weakening the polymer structure that makes up the capsule. This process helps to decrease bacterial virulence by allowing for the immune system to perform its action [70]. One interesting property of depolymerases is their wide bacterial range. In contrast to lysins, resistance against phage depolymerases can emerge due to modifications in polysaccharide composition of capsule, exopolysaccharides, or lipopolysaccharide [61]. New studies are currently emerging to ensure their safety and efficacy against capsulated *E. coli* in mice [69]. Moreover, it seems that some depolymerases eliminate biofilms, such as a depolymerase derived from a phage against *Staphylococcus epidermis*, which is an interesting application due to the difficulties of removing these formations. Depolymerases favor the penetration of the phage into the biofilm and the host cells due to its capacity for degradation of capsular polysaccharides [43]. Another group is the virion-associated peptidoglycan hydrolases (VAPGHs) as lysozymes, lytic transglycosylases, endopeptidases, or glucosaminidases. In contrast to endolysins that act in the final step of viral replication, VAPGHs can favour phage entry to the host by creating a hole through the cell wall of the host cell. With this, the phage can insert its tail and carry out the injection of its genetic material. An interesting example is gp49 from *S. aureus* phage phi11. Under normal conditions, gp49 is not necessary, but when the temperature or density fluctuates, this enzyme can improve infection. These enzymes are located almost anywhere in the phage, as they have been found in the tail, the head, the capsid, and the viral membrane [69,71]. Hydrolases are suitable to carry out their function in Gram-positive and Gram-negative cells because they are encoded by phages that attack both bacterial groups [19]. VAPGHs present several benefits that make them suitable as antimicrobial agents in therapeutic fields and also as food control or food decontaminants, especially in foods that undergo processes at high temperatures during manufacturing, as in dairy products. In addition, it has been shown that they can be useful against multiresistant bacterial strains [71]. Recent research proposed VAPGHs activity to treat plant diseases, such as those provoked by *Agrobacterium tumefaciens* [72]. Finally, to improve their bactericidal capacities, chimeric proteins can be created by combining several enzymes or by exchanging their functional domains. Some of these chimeric proteins have been used to treat diseases that are caused by *S. aureus*. Although few studies have been done to determine bacterial resistance emergence under VAPGHs treatment, bacterial resistance has not been reported so far [71].

Phage-derived enzymes are very useful and an attractive solution against bacterial infections. Synthetic biology allows for creating and modifying phage proteins to improve bacterial range spectrum, reduce bacterial resistance, and reduce immunogenicity. In addition, enzybiotics can be a great tool to be used against intracellular pathogens, where phages have difficulties to reach due to the lack of receptors for eukaryotic cells. Besides, phage-derived enzymes can be easily delivered into specific infection sites, acting locally in the infection and reducing side effects.

### 3.3. Phage Display

Despite the fact that natural bacteriophages have been studied to diminish bacterial resistance with success, some researchers have gone beyond this to find alternative antibacterial methods based on phages. This leads to phage modifications by gene engineering, the principal advantage of which is greater accuracy capacity [73]. However, there are more benefits of phage engineering, such as obtaining phage elements which are able to detect bacterial hosts, changing phage hosts, or promoting their action [74].

Phage display was developed in 1985 by George P. Smith [75]. It is a technique that is based on the expression of the phage cover of foreign peptides. The phage is exposed to the target (natural or synthetic) of interest until some of the peptides exposed in the phage bind specifically to that target. To carry out these processes, libraries of phage particles are commonly created by means of random peptides. These peptides are bound to components of the phage coat. Random oligonucleotides must be introduced at a specific location within the phage. The function of the library is to facilitate the search for the appropriate peptides through several screenings of ligands for each target. In addition, once the specific peptide has been recognized, it is possible to maximize its specificity and affinity. To check if the proteins that are expressed by the phage bind to the chosen target, a “biopanning” process is performed three to six times. This method consists of the immobilization of the target and its exposure to randomly selected peptide libraries. Then, less to more rigorous washes are made to eliminate phages that are bound nonspecifically to the target. Finally, once the phage has correctly attached to that target, different methods are used to separate it from the target and to sequence it until finding the specific peptide. It is usually said that phage display is a link between phenotype and genotype [76,77].

It is possible to use different types of bacteriophages for phage display, but the most advantageous are the filamentous ones, since they allow for genetic material expansion simply by increasing their filament size. The process of introducing genetic material into the phage does not damage its internal structures. These phages, which are usually temperate, do not kill bacterial cells to carry out their biological cycle and release new virions. Phage M13 is a typical bacteriophage used for this technology. In general, the most interesting gene in phage display is gene VIII, which codes for major structural proteins and is suitable for displaying short peptides and obtaining a high number of desirable molecules. In contrast, gene III codes for minor proteins and is more appropriate for expressing large peptides, although few copies of them will be obtained [78]. 

Phage display is an excellent tool for vaccine production, the development of new drugs, the study of protein–protein interactions, the selection and modification of substances of interest, the development of monoclonal antibodies with the desired specificity for therapeutic use, the creation of libraries of peptides and other substances, in epitope mapping (as antivenom), or the production of food as biocontrol. In the same way, it is a very useful technique to choose and isolate antibodies against desirable antigens or other targets, and to then create an antibody library [3,77,78]. Related to our subject of study, one application is the use of phage display against bacterial resistance. A typical response of bacteria when they are exposed to antibiotics is the secretion of enzymes. Particularly, they express β-lactamases, which hydrolyses the β-lactam ring with the aim of stopping antibiotic attacks, thus rendering the bacterial cells resistant to them. This resistance is generated as a result of the contact between bacteria and antibiotics presenting a β-lactam ring. β-lactam antibiotics have been widely used since their discovery. As a result, bacteria have been evolving, and, consequently, resistance has emerged. Through the implementation of phage display, this technique can be used to find peptides against β-lactamases enzymes by testing different peptide libraries [79]. Another example is the use of phage display against multiresistant strains of *L. monocytogenes*. These bacteria are usually transmitted by food and cause diseases such as arthritis and infections called listeriosis, which affect the central nervous system. As a result of the increase in antibiotic resistance, alternative methods have been sought to combat these diseases. One of these methods is the use of phage display and peptide libraries. After finding peptides that bind to the bacteria of interest (*L. monocytogenes*), peptides were isolated and it was checked if they presented microbicidal activities [80].

Similarly, the creation of new drugs opens many possibilities for future medicine. This is achieved by following the basic protocol of phage display but against therapeutic targets (e.g., specific points on which to act against pathogenic bacterial diseases). In addition, people under immunosuppression, such as HIV patients, transplant recipients, and pregnant women, are very susceptible to pathogenic infections. It is expected that more than 50% of patients with HIV will develop resistance to many of the current treatments [81]. Therefore, new alternative treatments should be proposed in order to solve this problem.

Moreover, phage display allows for the production of two main types of vaccines: phage display vaccines and phage DNA vaccines. On the one hand, phage display vaccines are based on a virion inside of which is the gene that codes for the antigen that is displayed, and they are more stable than phage DNA vaccines, when considering that the virion protects them. On the other hand, in phage DNA vaccines, inside of the virion there is DNA with the antigen gene that has been cloned in a eukaryotic cassette. As a result of these vaccines, there is a greater immune response than with conventional vaccines. Some of the investigations with these types of vaccines are directed against bacteria, autoimmune diseases, cancer, fungi, parasites, or even contraceptive vaccines [82]. There are also studies to use vaccines with the aim of preventing or reducing antimicrobial resistance. This could be innovative because they produce an immune response if the patient is exposed to a pathogen; as a result, the disease is avoided or weakened. In addition, if vaccination rates increase among the population, it can produce herd immunity, which protects unvaccinated people [13]. 

Like others methods, phage display presents some drawbacks that should be analysed. In the passage to a soluble medium, the binding capacity of the peptide to its target may be lost. In addition, peptide functions in vitro and in vivo can be different, with the risk of producing side effects in the patient. Peptides are also unstable due to proteolysis, and their ability to develop immune responses can be a problem for their application. Nevertheless, there are some techniques that are being developed to overcome these setbacks. Many of them are related to protein engineering or nanotechnology and are aimed at decreasing immunogenicity and increasing peptide affinity and half-life [83]. 

## 4. Conclusions

Multidrug-resistant bacteria are currently emerging for almost all the present-day antibiotics. Antibiotics are stable molecules exerting a selective pressure that allows for bacteria to evolve in order to escape, and new treatments should be proposed. Bacteriophages are a real alternative solution to this problem. Phages have different potential applications, starting from their use as bacterial killers in phage therapy, the use of their derivative enzymes, or their use in phage display. Phage therapy has been used for almost a century and it looks like a safe and effective treatment, although it is necessary to do more research to guarantee its safety in the short and long term. Very interesting and useful variants of phage therapy are emerging to enhance phage functions and to take advantage of them in many different areas. The large amount of possibilities due to the great diversity of phages, the use of phage cocktails, the combination with antibiotics, or promising phage display techniques allows for taking the most convenient approach for each scenario and to open new research areas to determine its advantages and disadvantages in each case. Surprisingly, there are also many new applications derived from phage therapy from which other fields can benefit. 

Phages appear to be a great solution not only as an alternative treatment against bacterial diseases (phage therapy or use of phage-derived enzymes) but also as interesting tools in the prevention (phage-delivered enzymes) and diagnosis (bacterial detection and typing). Despite the success that these phage-based treatments are expected to have, we are also facing a big concern: the lack of regulation in developed countries and the acceptance of the general public. Further research in this field will help to create regulatory and safety protocols that will lead to the general use of phages in the clinical and pharmaceutical fields. 

## Figures and Tables

**Table 1:** Summary of the main places where phages can be found: nature, urbanized places, and the human body.

**Phages in nature**	Soil
	Terrestrial subsurface
	Fresh water
	Ocean
	Oceanic subsurface
	Extreme environments: sea ice, algal flocks, hypersaline zones, etc.
**Artificial places**	Hospital and similar places
	Wastewater treatment plants
	Some areas under human impact
**Body of animals**	Digestive tract
	Vagina
	Respiratory and oral tract
	Skin
	Mucosal epithelium
